# Spatiotemporal dynamics of visitors to Jeju Island: Hotspot and spatial autocorrelation analyses using mobile phone data

**DOI:** 10.1371/journal.pone.0321694

**Published:** 2025-04-28

**Authors:** Kwang-Sub Lee, Jin Ki Eom

**Affiliations:** Railroad Policy Research Department, Korea Railroad Research Institute, Uiwang-Si, Korea; Zhongnan University of Economics and Law, CHINA

## Abstract

This study used mobile phone data to examine the spatiotemporal patterns and preferences of domestic visitors to Jeju Island, South Korea and integrated hourly floating population data to examine visitor characteristics by season, day of the week, time of the day, and inflow regions; identified hotspots at the census block level; and involved spatial autocorrelation analysis. The primary findings indicate that visitor numbers vary significantly by season, with summer and winter seasons attracting the highest number of tourists. The analysis revealed a concentration of visitors around Jeju Airport and the Jungmun Tourist Complex, suggesting that these are key hotspots. The inflow analysis underscores a dominant influx from major urban centers, particularly Seoul and Gyeonggi Province. The Global Moran’s Index confirmed a positive spatial autocorrelation across all seasons, with the strongest correlation observed in winter. The local spatial autocorrelation analysis identified significant clustering in hotspots, highlighting spatial interdependencies critical for marketing and infrastructure planning. This study uses big data analytics to advance the understanding of tourist behavior, offering critical insights for crafting sustainable tourism strategies and policies that align with demographic, temporal, and seasonal dynamics. The findings not only contribute to current empirical research but could also aid local governments and stakeholders in optimizing tourism management and enhancing visitor experiences on Jeju Island.

## Introduction

Jeju Island, located in the southwest region of South Korea, is the largest island and the most popular tourist destination in Korea. Halla Mountain and numerous small volcanic hills known as “oreum” in Korean can be found at the center of Jeju Island. The island also has numerous beaches, theme parks, and historical sites, making it a destination with a beautifully preserved natural environment and a concentration of various tourist facilities. The number of visitors to Jeju steadily increased from 7,578,301 in 2010 to over 15 million in 2019. In 2020, the number of tourists temporarily plummeted due to the pandemic but since then, has gradually increased following the easing of social distancing measures, reaching 13,889,502 in 2022, which is 91% of the pre-pandemic rate [1]. People visit Jeju primarily for relaxation and sightseeing (72.3%), leisure sports (9.6%), meetings and business (7.8%), and visiting relatives (7.8%); with most visits being for tourism and leisure activities [2]. Additionally, 99% of visitors in 2022 (13,803,058 visitors) were domestic tourists, and the utilization rate of rental cars was high at 77.8% [2].

In this context, an important aspect to consider is that an increase in the number of tourists does not always have a positive impact on the lives of residents [3]. The study of Liu et al. [4] revealed that while factors related to urban livability in China can aid tourism development, excessive tourism development could potentially threaten urban livability. Similarly, Vu et al. [5] found that while the spatiotemporal activities and movement patterns of tourists and residents differ in Paris, specific times (hours, seasons) or places (types of activities) can create extreme crowding hotspots, leading to issues of overtourism. Similar to these international examples, the steady increase in tourists and excessive use of rental cars in Jeju have caused traffic congestion, parking problems, and traffic safety issues, presenting challenges for the local government’s efforts to protect the pristine natural environment and develop sustainable tourism destinations. Consequently, the local government of Jeju legislated the management of total vehicle volume and announced a rental car control plan including restrictions on the number of tourist rental cars and new registrations [6].

Despite the overtourism and safety issues, the tourism industry substantially impacts various sectors (e.g., social, cultural, economic, and environmental) in the region [7]. Accordingly, continuous research has been undertaken in several study areas, including forecasting tourism demand [8,9], analyzing the spatiotemporal visitation patterns of tourists [10–12], changes in tourist behavior [13,14], and interconnections between the tourism industry and local tourism development [7].

Tourism activities inherently encompass spatiotemporal phenomena, making it crucial to understand the spatiotemporal behavior patterns of tourists [15–17]. Clustering [18,19] and hotspot analysis are commonly used to study the spatial distribution of tourist sites. Clustering analysis poses challenges in statistically proving suitability, leading to the use of hotspot analysis to validate spatial autocorrelation, however its limited because it only considers spatial aspects [10].

Furthermore, the distribution of hot and cold spots at tourist sites can vary depending on the characteristics of tourists (e.g., nationality, age, and gender) and time-related factors (e.g., season and hours of the day) [10]. Tourist sites also interact with neighboring areas and share continuities, necessitating a comparison of overall preferences across multiple sites rather than single sites [20].

The use of big data containing spatiotemporal information has increased in tourism research [7,10,21]. However, some of these data are sparse and bias, and the data collection process can be time-consuming [10]. This highlights the need for advanced analytical techniques and robust methodologies to mitigate these limitations and fully exploit the potential of big data in tourism research.

Against this background, the current study aimed to analyze the seasonal characteristics and preferred hotspots of domestic visitors to Jeju Island and identify regional clusters through spatial autocorrelation analysis at the census block level, by examining hourly floating population information data obtained from mobile phone records. We compared the characteristics of external visitors to Jeju Island by season, weekday/weekend, and time of the day, focusing on the following aspects: (1) number of external visitors, (2) visitor demographics by age, (3) origins of the visitors (home), (4) preferred destinations (hotspots) at the census block level, and (5) spatial relationships and distribution patterns assessed through both global and local spatial autocorrelation analyses.

The novelty and contributions of this research can be summarized as follows: (1) using big data from mobile phones to enhance the understanding of tourist behavior by season, weekday, and time of the day; (2) using hotspot analysis to compare preferred destinations by time, day, and season by considering a more detailed spatial unit (census block group) than traditional administrative areas; and (3) identifying visitor clusters and examining their mutual relationships and seasonal distribution patterns through statistically valid spatial autocorrelation analysis.

The outcomes of this research, based on a spatial understanding of the characteristics and preferences of external visitors, could inform the development of tourism marketing strategies tailored to demographic, temporal, and seasonal factors. These strategies could facilitate the development of tourism infrastructure that ensures visitor safety while protecting the natural environment, fostering tourism industry growth, and ultimately serving as foundational data for future transportation and urban planning on Jeju Island.

## Literature review

### Studies on spatiotemporal visit patterns of tourists

Huang et al. [17] emphasized the importance of spatiotemporal analysis of tourist behaviors under activity-based constraints in understanding three principal dimensions of the tourism experience, namely, time, space, and activity. Understanding the movement characteristics of tourists by examining the spatial attributes is essential because of the inherent spatial phenomena of tourism activities.

Studies have frequently utilized clustering analysis or hotspot analysis to examine spatial distributions. Clustering analysis classifies spatial distributions based on trends using unsupervised machine learning algorithms, which inherently lack the ability to statistically validate their suitability. In contrast, hotspot analysis offers an advantage in this regard as it can be used to statistically prove spatial autocorrelation and overcome the traditional statistical pitfalls based on the principle of independence, thus explaining spatial heterogeneity effectively [22].

For example, Karagoz et al. [23] analyzed the relationship between natural, cultural, and historical tourist attractions and tourist flows in Turkey using mapping analysis, global and local Moran’s I, and both classical regression and spatial regression models. Kang et al. [24] employed local indicators of spatial association (LISA) using geographic information systems (GIS) to analyze the network of attractions within tourist sites, demonstrating the presence of multiple anchor points (attractions) within tourist destinations. Further, Xu and Wang [25] collected data on tourist attractions in the Anhui Province of China and applied GIS spatial analysis techniques to examine the distribution characteristics of these attractions. Huang et al. [17] presented a methodology to identify behavioral clusters based on the spatiotemporal characteristics (path length, travel time, etc.) of tourists visiting Ocean Park in Hong Kong, using portable global positioning system tracking data and survey responses. In addition, Magdolen et al. [26] conducted a survey among residents of Berlin and Munich in Germany to identify and compare the types of leisure activities associated with long-distance leisure travel.

Studies have also been conducted on tourist patterns related to specific times, such as vacation seasons, or specific places. Liu et al. [12] analyzed holiday season travel patterns using smart card data from the Shenzhen Metro system and points of interest data to study traffic patterns during the Chinese Spring Festival season.

Additional research includes analyses of travel destinations [27,28], factors influencing tourists’ intentions to revisit [14], the demographic and travel characteristics of tourists [29], and satisfaction with specific tourist sites [30]. These studies provide insights into the complex dynamics of travel behavior and tourist preferences, which are crucial for developing targeted marketing strategies and enhancing visitor experiences.

### Studies on tourists and tourist destinations using big data

Recently, big data have been actively utilized in various fields. In tourism research, meaningful patterns, trends, and insights are extracted from big data to enhance the competitiveness of tourist destinations and improve visitor experiences. Big data offers the advantage of easily identifying the sparsity and spatiotemporal characteristics of data, particularly beneficial for studying tourism travel patterns. Studies utilizing Google Trends big data [31] as well as geotagged data, mobile telecommunications, and credit card data are increasingly common in this research area.

Studies utilizing geotagged and social media data to analyze tourists’ movement patterns or preferred destinations have revealed insightful trends. Several studies analyzed location-tagged photos uploaded to the photo-sharing site Flickr to study the behaviors of tourists visiting tourist destinations in Melbourne, Australia [32], the USA [33] in New York City [34], and Beijing [35]. Maeda et al. [36] compared the patterns of tourist spots visited by foreign versus domestic tourists in Japan, based on geotagged social media data acquired from Twitter and Foursquare. Twitter data were also used by Hasnat and Hasan [11] to classify tourists and residents, who then employed clustering methods to analyze the spatial patterns of destination choices among tourists visiting Florida. Further, van der Zee et al. [27] applied network analysis methodologies using user-generated content from TripAdvisor to analyze the relationship between tourist behaviors and tourist destinations in Antwerp, Belgium.

Research utilizing big data sources such as mobile phone data has been valuable in addressing the vulnerabilities of traditional tourism statistics, offering timely and effective insights for tourism strategies, policies, and marketing. For example, Qin et al. [7] utilized mobile phone call detail record data and real-time location services integrated into mobile phones to analyze the distribution of tourist hotspots, destinations, origins, and movements in Beijing, demonstrating the utility of mobile big data analytics in providing real-time information on tourist behaviors. Park et al. [16] applied a trajectory data mining approach based on mobile tracking data to identify the characteristics of tourist destinations visited by foreign travelers in Korea. Similarly, using mobile data Hwang et al. [10] analyzed the movement behavior patterns of foreign tourists visiting Jeonju City in Korea.

In addition to movement patterns, mobile and credit card big data have been used to study tourist destinations, develop models for tourism activation, analyze tourists’ consumption behaviors and patterns, and determine the locations of accommodation facilities. Raun et al. [37] utilized mobile tracking data to present a methodology for estimating tourist destinations in Estonia from spatial, temporal, and compositional perspectives.

Oh and Kang [21] used big data from telecommunications and credit card companies to diagnose tourism activation at the municipal level and identify key factors that promote tourism activation. An and An [38] combined mobile telecommunications and credit card data with GIS analysis to study the movement paths and consumption patterns of tourists visiting Yongin City in Korea. Silva et al. [39] combined traditional statistical data with big data from online booking services to analyze major tourist sites in Europe in terms of tourism prevalence, seasonality, and intensity. These studies highlight the extensive potential of big data in enhancing the precision and effectiveness of tourism research and development strategies.

## Data and methodology

### Mobile phone data

In Korea, the mobile phone penetration rate reached 94.2% in 2022, indicating that nearly all citizens carry mobile phones, making it possible to gather almost census-like data and determine users’ spatiotemporal locations [40]. This study utilized floating population data obtained from SK Telecom (SKT) telecommunication company. SKT has the largest number of customers (about 50% of all customers) among the three telecom companies in Korea [41]. SKT weights the number of SKT users based on their market share in each region (using the distribution of users by gender and age) so that it represents the entire population of mobile phone users in Korea. This is a unique advantage that SKT floating population data has, which is not found in any other country’s mobile phone data.

SKT’s mobile-based floating population data measure the population that stays (or is active) in each spatial area on an hourly basis, conceptually similar to a snapshot. Mobile phones continuously communicate with cell tower transceivers as long as they are not turned off, regardless of whether they are being used for calls, texts, or apps. Therefore, the location of mobile phone users can be accurately determined at any time and place. The floating population data were generated by first collecting communication connection records from each base station and then determining the number of people within a virtual 50 m grid, subsequently normalized to the total population. These data were categorized by sex, age group, and time period, and included home location details that indicate the inflow location based on actual residential locations. The population within the 50 m grids was further aggregated into the spatial extents of the census blocks. The actual residential location was estimated based on the nighttime locations detected for mobile phone users recorded between 11 PM and 4 AM at the same location for at least 15 days within the past month. Therefore, unlike usual legal population statistics, which are based on registered residences and often do not match the actual residences, mobile-based floating population data more accurately reflect both the actual residences and the locations at the time of measurement. This distinction is crucial for providing a more precise and dynamic understanding of population movement and density, which is essential for various urban planning and policymaking processes.

As shown in Table 1, the floating population data comprise information on the date, time, geographic coordinates of the grid cell center, census block group code the grid cell belongs to, inflow location (home) code, and number of floating population (including distinctions by sex and age from teenagers to those over 70 years in 10-year increments).

**Table 1:** Structure of mobile phone data.

Variable		Description
Date		Date
Time		Time of day
X-coordinate		x-coordinate of a grid cell center point
Y-coordinate		y-coordinate of a grid cell center point
Census block		Code of census block group
Home		Code of inflow location (home location at city, county, and district level)
Floating population	Man in his 10s	Number of floating population by sex and age with the same inflow location while active in the grid cell
	Man in his 20s	
	Man in his 30s	
	…	
	Man over 70s	
	Woman in her 10s	
	Woman in her 20s	
	Woman in her 30s	
	…	
	Woman over 70s	

The SKT mobile original data used in this study contained weekly seasonal floating population information for Jeju Island for one week each in January (1/10–1/16), May (5/16–5/22), July (7/25–7/31), and October (10/24−10/30) of 2022. This study focused on domestic visitors from outside Jeju Island; therefore, data for cases where the home location was not on Jeju Island were extracted for analysis. The volume of data varied by date and time, with the highest number of visitors recorded at 10 AM on July 30th, totaling 4,056,991 records, of which 2,683,451 were from external visitors. It should be noted that one record does not represent one person but represents the population counted at a specific time in each census block group on Jeju Island, with the same home location, categorized by sex and age group.

### Spatial autocorrelation analysis

Spatial autocorrelation analysis is an important approach in geographical data analysis, which assesses the relationships between data within a geographic space. This type of analysis helps to determine how observations in one area relate to those in adjacent areas, identifying whether the interconnections between spatial data follow a specific pattern rather than occurring by chance.

The existence and strength of spatial autocorrelation are commonly measured using two indices: Moran’s I and Geary’s Ci. In this study, we utilized the Moran’s I statistic devised by Moran [42]. The Moran’s I index is divided based on two types of analyses: global and local. Global analysis measures the overall level of spatial autocorrelation for a dataset and provides a single summary statistic. The formula for Global Moran’s I is as follows:


$$I_{global}=\frac{n\sum \sum w_{i,j}\left(x_{i}-\bar{x}\right)\left(x_{j}-\bar{x}\right)}{W\sum {\left(x_{i}-\bar{x}\right)}^{2}}$$ (1)

where:

*n* is the number of spatial units,

*W*is the sum of all spatial weights,

$w_{i,j}$is the spatial weight between regions *i* and *j*,

$x_{i}$and $x_{j}$ are the attribute values for regions *i* and *j*,

$\bar{x}$is the mean of the attribute value *x*.

Specifically, the Global Moran’s I value is a coefficient, which is obtained by measuring the similarity of attribute values between adjacent neighborhoods, illustrated through a spatial weight matrix that shows the spatial adjacency between areas. The Global Moran’s I index ranges between -1 and +1:

- A value close to ‒1 indicates negative spatial autocorrelation or dispersed spatial patterns.
- A value close to +1 indicates positive spatial autocorrelation or highly formed spatial clusters.
- A value of 0 indicates a random distribution or that the spatial correlation is statistically insignificant.

However, Global Moran’s I has limitations in pinpointing where clusters occur specifically or if the clustering is concentrated in only a part of the study area. To address these limitations, Anselin [43] developed the LISA method. It calculates a statistic for each area and interprets a positive (+) spatial autocorrelation if the statistic for an area is similar to the weighted average of its neighbors’ statistics. The formula for calculating Local Moran’s I is as follows:


$$I_{i}=z_{i}\cdot \sum_{j=1}w_{i,j}z_{j}$$ (2)

where:

$z_{i}$and $z_{j}$ are deviations from the mean for regions *i* and *j*,

$w_{i,j}$is the spatial weight between regions *i* and *j*.

Local Moran’s I values can visually represent spatial correlations in four distinct categories on a LISA cluster map. The first type, high-high, refers to areas where both the focal area (in this study, a census block group in Jeju Island counting external visitors) and its neighbors have high values, indicating that areas with high values are adjacent to each other. The low-low type describes situations where areas with low values are adjacent to other areas with low values. The low-high type indicates areas with low values next to areas with high values. Conversely, the high-low type refers to areas with high values adjacent to areas with low values. Therefore, the high-high and low-low types show local spatial clustering of similar values. The LISA cluster map allows for the visual identification of cluster locations, providing critical insights regarding regional clusters of seasonal visitors, illustrating both the concentration of visitors and their spatial correlation with the surrounding areas, and how different seasons impact the visitor flow in each region.

In this study, we analyzed the spatial patterns of external visitors to Jeju Island using exploratory spatial data analysis (ESDA) from both global and local perspectives. ESDA is an open-source Python library for the exploratory analysis of spatial data [44]. As a sub-package of PySAL (Python Spatial Analysis Library), it is a collection of GIS-based techniques useful to describe and visualize spatial distributions, identify locational or spatial outliers, and determine spatial association patterns [45,46].

## Results

### Analysis of characteristics of external visitors by day, age, and inflow point

#### External visitors’ seasonal and day-specific characteristics.

**Fig 1:**
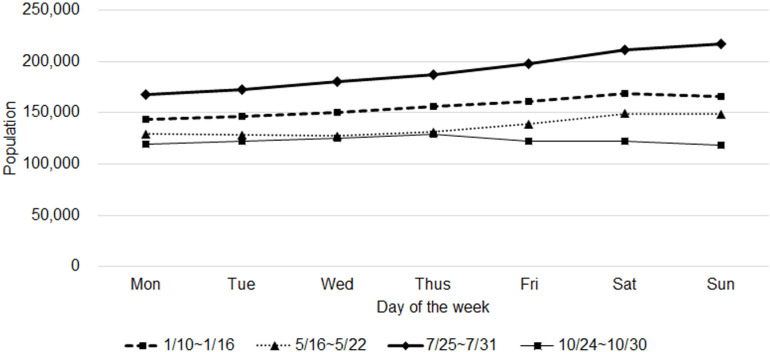
Comparison of total incoming population (10 AM).

When comparing the external visitor population data at 10 AM for one week per season, the summer months (July) consistently showed the highest number of visitors across all days, followed by winter (January), spring (May), and fall (October) in descending order (Fig 1). The day with the highest visitor count was July 31st, a Sunday, with 216,908 visitors, and the day with the lowest was October 30th, also a Sunday, with 118,158 visitors. The high visitor numbers in July and January coincide with the vacation and holiday seasons.

The average number of visitors on weekdays (Monday to Friday) in July was 181,108, while the weekends (Saturday and Sunday) saw an average of 214,094 visitors, showing an 18% increase during weekends. January, May, and July generally saw higher weekend visitor numbers compared to weekdays, whereas October showed little variation between weekdays and weekends. This seasonal and weekly variability highlights the impact of vacation timings and Jeju Island’s natural attractions such as beaches and mountains on tourist inflow.

#### External visitors’ characteristics by age groups.

After categorizing the age groups from teenagers through those in their 70s in 10-year increments, we compared the external visitor populations at 10 AM on Wednesdays (as a representative day of the week) and Saturdays (as a representative day of the weekend) across different seasons. The age group with the fewest visitors on a weekday was those in their 70s, with 9,944 visitors on Wednesday, May 18th. Similarly, the same age group had the fewest visitors on a weekend, with 10,192 on Saturday, October 29th. The highest number of external visitors on both weekdays and weekends was among teenagers: 54,354 on Wednesday, July 27th, and 64,445 on Saturday, July 30th, respectively. These data indicate that teenagers constitute the predominant age group visiting Jeju Island, particularly during the month of July, which coincides with the summer vacation period.

Seasonal variations showed distinct patterns in the age-specific visitor populations on weekdays compared to weekends. In October, the visitor counts for all age groups were lower on Saturdays compared to Wednesdays, with teenagers and those in their 70s experiencing the largest declines of 44.7% and 40.6%, respectively, and those in their 50s and 40s seeing decreases of 15.7% and 19.1%, respectively. Conversely, in May, the visitor counts for all age groups increased on Saturdays compared to Wednesdays, with increases ranging from a minimum of 20.7% among those in their 20s to a maximum of 39.7% among those in their 50s. This indicates a significant shift in visitor dynamics influenced by factors such as public holidays or seasonal attractions.

Using October (fall), when the overall external visitor population was at its lowest, as a baseline for comparison, we observed intriguing seasonal variations in age-specific visitor populations between weekdays (Wednesday) and weekends (Saturday). On Wednesdays, visitor counts across all age groups in May showed a decrease compared to October, with teenagers experiencing the most significant drop of 51.6% and those in their 30s showing the smallest decrease of 23.1%. In contrast, other seasons (summer and winter) showed trends of generally maintained or increased visitor counts across all age groups compared to autumn. Notably, in winter, there was a 49% increase in visitors in their 20s compared to autumn. When comparing weekend visitor populations by age group, taking October as the reference point, there were increases across all age groups in other seasons, with particularly significant increases in January and July. Relative to the counts in October, the surge in visitor numbers in July was exceptionally high, increasing as follows by age group: teenagers, 314.0%; those in their 20s, 200.8%; and those in their 70s, 237.5%. The substantial increase in summer likely reflects the seasonal appeal of Jeju Island’s outdoor and recreational activities, which are particularly attractive to younger visitors during school vacations.

**Fig 2:**
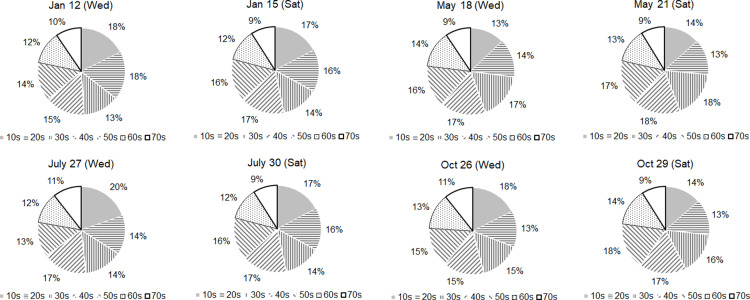
Comparison of external visitors by age on weekdays and weekends at 10AM.

The comparative results of the age group composition of seasonal visitor populations on Wednesdays and Saturdays are presented in Fig 2. Teenagers consistently accounted for 13%−20% of the visitors across all seasons, with lower proportions during the school months of May and October and higher proportions during the vacation months of July and January. It was also observed that the weekday proportions of teenage visitors were similar to or higher than the weekend proportions within the same season (e.g., July 27th (Wed) 20% vs. July 30th (Sat) 20%; October 26th (Wed) 18% vs. October 29th (Sat) 14%). Visitors in their 20s and 30s showed proportions between 13% and 18%. Visitors in their 40s (15%–18%), 50s (13%–18%), 60s (12%–14%), and 70s (9%–11%) displayed relatively stable visitation rates across the seasons, indicating less sensitivity to seasonal changes.

#### External visitors’ characteristics by inflow point.

Based on the home locations of external visitors active in Jeju Island at 10 AM, the analysis revealed that residents from Seoul and Gyeonggi Province consistently comprised the largest portion of visitor demographics across all seasons. Combined, visitors from these two regions accounted for approximately 50% of all external visitors to Jeju Island, with the lowest proportion in October at 44% and highest in July at 52%. Other provinces accounted for only 1%–7% of the visitors. The overwhelming proportion of Seoul and Gyeonggi residents can be primarily attributed to the fact that these areas together comprise approximately 44% of South Korea’s total population. Although Gyeonggi Province has a larger registered population (26%) than Seoul (18%), similar visitation rates to Jeju from these regions suggest a relatively higher travel frequency from Seoul residents. This indicates not only the demographic influence on travel patterns but also possibly higher disposable incomes among Seoul’s populace.

**Fig 3:**
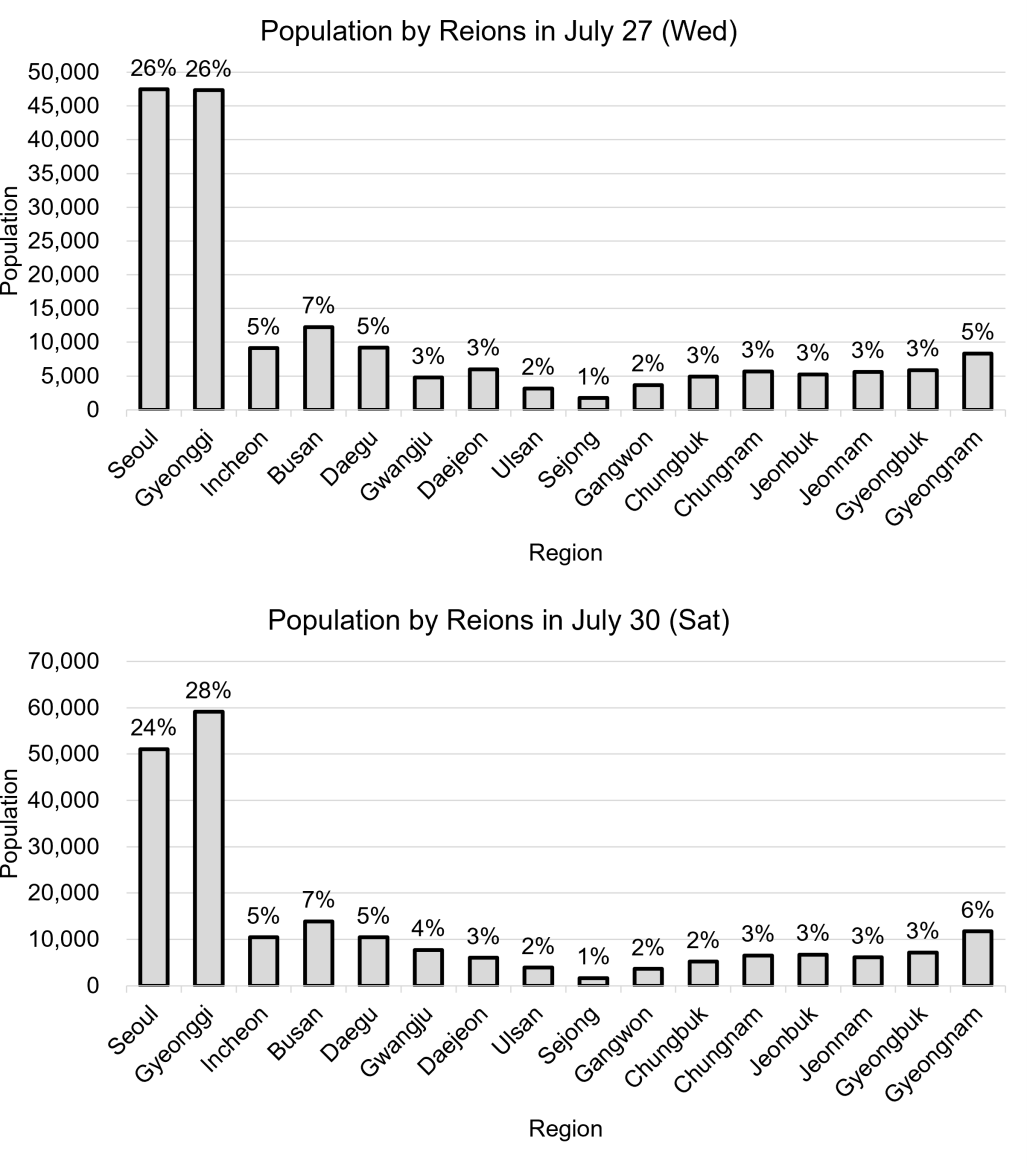
Comparison of external visitor rates at 10AM by residence on Wednesday (top) and Saturday (bottom) in July.

The composition of visitor origins (residence) for July is displayed in Fig 3 for both Wednesday and Saturday. There was no significant difference in the source of visitors, with both periods showing the highest proportions from Gyeonggi Province and Seoul. On Saturday, July 30th, there were slightly more visitors from Gyeonggi Province, totaling 59,131 (28%), compared to 50,737 visitors from Seoul (24%). This analysis provides valuable insights for regional tourism planners and marketers in Jeju Island to tailor their strategies effectively.

**Fig 4:**
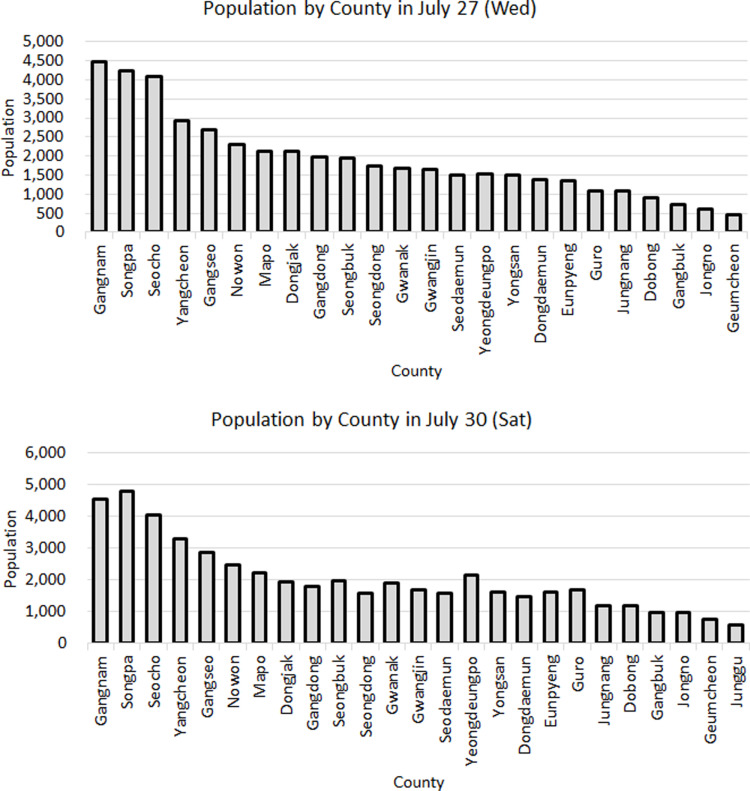
Comparison of the ratio of Seoul residents by county (Wednesday (top) and Saturday (bottom) at 10AM in July).

When analyzing the inflow of visitors from Seoul to Jeju Island at 10 AM on both a Wednesday and a Saturday in July, a detailed county-level analysis revealed that the highest proportion of visitors came from the wealthiest districts in Seoul, known as the “Gangnam 3 Counties” (Gangnam, Songpa, and Seocho), as shown in Fig 4. On Wednesday, Gangnam led with 9.42%, followed by Songpa at 8.90%, and Seocho at 8.58%, showing a significant number of visitors. On Saturday, the pattern was slightly different, with Songpa having the highest visitor rate at 9.40%, followed by Gangnam at 8.94% and Seocho at 7.90%.

**Fig 5:**
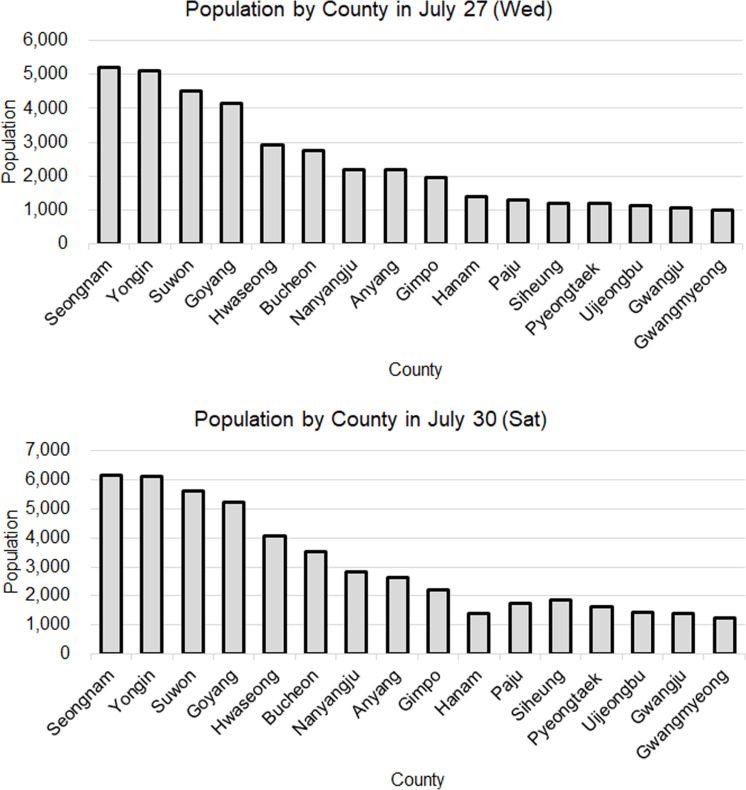
Comparison of the ratio of Gyeonggi residents by county (Wednesday (top) and Saturday (bottom) at 10AM in July).

Analyzing residents from Gyeonggi Province to Jeju Island, the residents of Seongnam, Yongin, Suwon, and Goyang consistently showed high visitation rates (each over 10%) across weekdays, weekends, and all seasons. For instance, there was a significant influx on Saturday from Seongnam (6,151 visitors, 12.53%) and Yongin (6,102 visitors, 12.43%), as shown in Fig 5. Interestingly, in January, Yongin had the highest weekday visitor rate at 12.20%, whereas Suwon had the highest weekend visitor rate at 12.37%. Apart from the four cities (Seongnam, Yongin, Suwon, and Goyang), which accounted for the majority of visits, the other 26 cities in Gyeonggi Province had visitor proportions ranging from 2.57% to 7.82% on weekdays and 2.53% to 8.31% on weekends. Notably, the residents of Hanam City exhibited a unique trend where the proportion of visitors to Jeju decreased on weekends compared to weekdays. Furthermore, considering the population composition of Gyeonggi Province, the visitation rates to Jeju Island generally reflect the population distribution within the province. However, Seongnam City’s visitation rate was relatively high, whereas that of Suwon City was comparatively low.

### Preferred destination (hotspot) analysis

**Fig 6:**
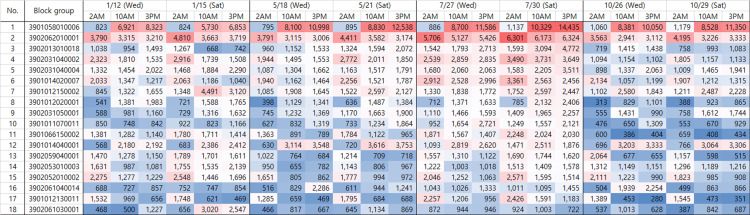
Preferred destinations (18 hospots) of external visitors by season and time of day.

Based on hourly mobile phone data, we analyzed the preferred destinations, or hotspots, of external visitors to Jeju Island by census block group. First, to represent weekdays and weekends, we selected Wednesday and Saturday for each season. We then calculated the hourly number of external visitors during the night (2 AM), morning (10 AM), and afternoon (3 PM) periods, selecting block groups with at least 2,000 visitors per hour. The data were sorted based on the population activity at 3 PM on Saturday, July 30th (Fig 6). The column on the left (No.) indicates the ranking and that labeled block group shows the identification number of the block groups; the color grouping indicates block groups from a similar location. For example, block groups 2, 16, and 18 are located slightly west of the southernmost center of Jeju Island, near the Jungmun Tourist Complex, whereas block groups 4, 5, and 9 are located slightly north of Jungmun. Block groups 13–15 are located in the central region of Seogwipo City at the southernmost center of Jeju Island, and block groups 7, 8, and 10 are located around the northwestern coast of the island. Additionally, in the hourly population data, a deeper red color signifies a higher number of hourly visitors, whereas a deeper blue color indicates fewer hourly visitors.

Throughout all seasons, on both weekdays and weekends during the daytime, the area with the highest number of external visitors, as expected, was Jeju Airport (block group 1). Excluding block group 1, the place with the highest number of external visitors at all times of the day and in all seasons was Jeju Jungmun (block group 2), a large tourist resort located in the central-south region of Jeju Island. This area is renowned for its accommodations, leisure facilities, and conventions, making it a top destination for tourists. The hourly number of external visitors here ranged from a minimum of 2,941 (on Wednesday, October 26th at 10 AM) to a maximum of 6,324 (on Saturday, July 30th at 3 PM), maintaining a consistent flow of at least 2,000 visitors per hour throughout the year and across the day and night.

Block groups 3–7 attracted the highest number of visitors in all time slots during the summer. The main tourist attractions in these areas are Seopjikoji, famous for its rape flowers and coastal cliffs (block group 3); Jeju Shinhwa World, a large complex with a theme park and resort facilities (block group 4); O’sulloc Tea Museum, famous for its green tea (block group 5); Hamdeok Beach (block group 6); and the Equestrian Park (block group 7). Block group 3 experienced an increase in visitors in the afternoon compared to the morning, likely because of its location in the southeastern part of Jeju Island, which requires significant travel time from other areas. Block groups 4 and 6 consistently saw a high number of visitors across all four seasons, with minimum external visitor counts of 1,094 and 1,040 per hour, respectively. In particular, block group 4 had a high visitor count across all hours in January, May, and July. Meanwhile, block group 5 had a significant number of visitors during the winter and summer seasons. Block group 6 typically had more visitors at night than during the day, suggesting a focus on accommodation, although it also saw increased activity during the day in the summer months.

In contrast, block groups 7–10, which feature outdoor leisure spaces and facilities such as beaches, equestrian parks, ranches, and small mountains created by a volcano (oreum) had more visitors during the daytime than at night. Additionally, block groups 3, 6, and 7 had fewer visitors in the fall and winter. Block groups 8–11 experienced a notable concentration of external visitors during the daytime in July, with significantly fewer visitors in other seasons and times of the day. Particularly, Block group 10, which includes Hyeopjae Beach and Biyang Island, had a concentration of visitors exclusively in the summer months. This seasonal visitor pattern underscores the appeal of these outdoor recreational sites, especially during periods of warmer weather when beach and island activities are most popular.

Block group 12, the EcoLand Theme Park, where visitors can explore the forest by a steam-powered train, saw a steady influx of external visitors during daytime hours across all seasons, due to the lack of accommodation facilities in the area. Block groups 13–15 had relatively fewer external visitors in May and October. Particularly during the daytime in May, these areas saw fewer than 1,000 visitors per hour. Notable attractions in these regions include the Jeju World Cup Stadium and the Seogwipo Olle Market. Block group 16, near the Cheonjiyeon Waterfall in Seogwipo, had very few visitors in January, with visitor numbers only concentrated on May 18th (Wednesday at 3 PM) and October 26th (Wednesday at 10 AM and 3 PM). This pattern likely reflects the timing of group visits by middle- and high-school students as part of field trips. Block group 17, where Gwakji Beach and coastal cafés are located, showed a higher number of visitors during the early morning hours, indicating the substantial use of local accommodations. In contrast, block group 18, which has the columnar joints near the coast saw visitor concentrations only during specific times—on January 15th (Saturday), with 3,020 visitors at 10 AM and 2,547 at 3 PM—suggesting that this location attracts visitors primarily during select periods.

### Spatial autocorrelation analysis

To determine whether the spatial distribution patterns of external visitors to Jeju Island were statistically significant, we first conducted a global spatial autocorrelation test for the floating population of external tourists at 10 AM on Saturdays for each season. As shown in Table 2, the Moran’s I values ranged from 0.1805 (October) to 0.3119 (January), indicating a positive spatial autocorrelation across all seasons. The p-values for Moran’s I at the 95% confidence level were all statistically significant at 0.001. This result confirms that there is a positive spatial autocorrelation. Furthermore, the Moran’s I values decreased from winter to spring to summer to fall, indicating stronger clustering and higher similarity between adjacent areas during winter.

**Table 2:** Results of global spatial autocorrelation analysis.

Date	Number of census blocks	Moran’s I	Z-value	p-value
1/15 (Winter)	1,380	0.3119	18.7881	0.001
5/21 (Spring)	1,384	0.2094	14.4896	0.001
7/30 (Summer)	1,386	0.1982	13.7597	0.001
10/29 (Fall)	1,383	0.1805	12.0761	0.001

**Fig 7:**
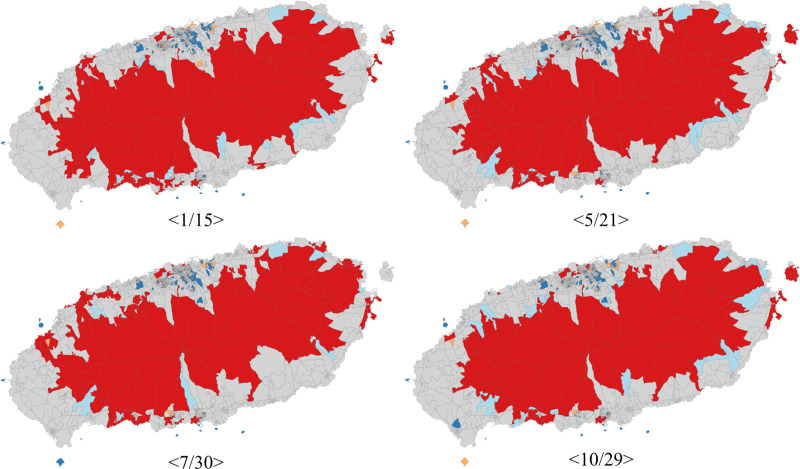
LISA Cluster Map (high-high: red, high-low: orange, low-high: light blue, low-low: blue, and not significant: gray) using ESDA, an open-source Python library.

Next, we performed a local spatial autocorrelation analysis. The cluster map of census block-level external visitor counts in Jeju Island (based on Saturday at 10 AM) obtained through LISA is shown in Fig 7, which is generated using ESDA open-source Python library. It is important to note that the boundaries and sizes (areas) of the block groups are based on the registered residential population of approximately 500 people. Consequently, the block groups in the central region of Jeju Island, where Halla Mountain is located, are relatively large in spatial size. In contrast, the block groups in the densely populated central-northern (central Jeju City) and central-southern (central Seogwipo City) areas of the island are significantly smaller in size.

When examining the seasonal distribution patterns, the high-high type was consistently concentrated around the central part of Jeju Island in all four seasons. This indicates that the visitor numbers in these block groups are positively correlated with those of the adjacent block groups and are significantly clustered. Seasonally, the variations in high-high type distribution appeared primarily in the northwest at Aewol-eup, the northeast at Jocheon-eup, the southwest at Andeok-myeon, and the southeast at Namwon-eup. In addition, all 18 hotspots listed in Fig 6 were of the high-high type.

Census block groups with high-high type were the most numerous in January, totaling 276, showing a broader distribution in the southwestern and southeastern regions. In May, the spread of this type extended to the northwestern and northeastern areas. By July, this distribution had expanded extensively to the northwest and northeast, resulting in a decrease in the number of block groups in the southwest and southeast. In October, the count of high-high type block groups was at its lowest at 234, and coastal areas in the northwest and northeast showed a tendency to fall outside the high-high type block groups. Compared to the other types, the number of block groups classified as the low-low type was the smallest in all seasons, predominantly concentrated in the north-central region around downtown Jeju City and the south-central region around downtown Seogwipo City. In May, the count of this type was the lowest at 66, especially clustered around downtown Jeju City and the central-northern areas. The season with the highest number of low-low type block groups was fall (October), with 78 block groups. Areas classified as high-high and low-low types indicate the presence of spatial autocorrelation. In contrast, the areas classified as high-low and low-high, which represent spatial anomalies, were predominantly found in the north-central region (near downtown Jeju City) and on the periphery of the high-high areas. The number of block groups classified as high-low type ranged from 219 to 251 and low-high type ranged from 867 to 919 across different seasons. These two types do not appear prominently in Fig 7 because they are found in areas densely populated by Jeju residents, leading to very small block group sizes.

## Conclusion

Although studies on the characteristics of tourist destinations and visitors have been conducted using various data, few studies have utilized the entire population of mobile phone users visiting a destination. In this study, we preprocessed big data (from one week in each of the four seasons with up to four million records per hour) representing all mobile phone users visiting Jeju Island. Using hourly floating population data, we analyzed the spatiotemporal characteristics of destination and preferred hotspots in temporal and seasonal contexts to present empirical and policy-applicable results that will serve as a benchmark for future analyses of other tourist destinations.

Specifically, we examined the spatial autocorrelation of visitor distribution and identified clusters of preferred areas as part of a detailed analysis of visitor behavior on Jeju’s Island major tourist sites and their surrounding areas. Regarding the seasonal daily characteristics of external visitors to Jeju Island, we found the highest number of visitors during the summer and winter months, indicating concentrated tourist activity during Korea’s vacation seasons. The visitation rates on weekends were approximately 18% higher than those on weekdays, reflecting a preference for weekend travel. When assessing visitors by age, teenagers were the most active group visiting Jeju in the summer. The frequency of visits decreased with increasing age. An analysis of visitor origin showed that approximately 50% of all visitors came from Seoul and Gyeonggi Province, largely because of the high population density and relative affluence of these regions. Many visitors came from Seoul’s wealthy districts—Gangnam, Songpa, and Seocho—indicating that economic factors significantly influence travel decisions. An analysis of preferred destinations based on census block-level hotspots revealed that aside from Jeju Airport, the Jungmun Tourist Complex was the most popular area among external visitors. Seasonal trends showed an increase in the number of visitors to regions offering beach and outdoor leisure activities during the summer.

Global spatial autocorrelation analysis revealed spatial autocorrelation across all seasons, with particularly strong clustering between neighboring areas in the winter. Local spatial autocorrelation analysis identified visitor-favored regions and their interactions with surrounding areas, offering vital foundational data for formulating regional tourism strategies.

This study highlights distinct tourist characteristics and patterns on Jeju Island:

- Seasonal visitor distribution: Jeju Island has a significant concentration of tourists during the summer and winter months, aligned with school holidays and vacation seasons, thereby attracting many family-oriented tourists.
- Age distribution of tourists: The age distribution of tourists, especially teenagers, shows seasonal variation. Visitor numbers are lower in May and October during school terms, with a relatively higher proportion of teenage visitors during weekdays in October, coinciding with school trip seasons.
- Regional influx patterns: Jeju Island’s distinctive feature is its high influx of visitors from densely populated regions, such as Seoul and Gyeonggi Province, influenced by economic factors.
- Hotspot analysis: Tourists on Jeju Island tend to concentrate around specific hotspots such as Jeju Airport, the Jungmun Tourist Complex, and areas rich in natural and leisure spaces and facilities.
- Spatial autocorrelation analysis: The results confirm positive spatial autocorrelation in all seasons on Jeju Island, with strong spatial clustering during the winter season, emphasizing Jeju’s unique seasonal visitor concentration.

This study has significant implications for tourism policy and infrastructure planning in Jeju Island. The spatial autocorrelation analysis results shed light on the concentrations of tourists in specific areas. This information can guide improvements in transportation systems and traffic safety, the optimization of tourist services, and measures to protect the environment. Additionally, understanding changes in visitor preferences across seasons can inform effective marketing strategies and event planning, contributing to the development of a sustainable tourism model. This analysis can also provide valuable insights for policymakers in other tourist destinations who are developing customized tourism strategies in their respective regions.

The mobile phone data used in this study is weighted based on SKT’s 50% market share in Korea and regional gender/age distribution of users to represent the entire mobile phone user population in Korea. This is a unique advantage that only SKT mobile data has, which cannot be found in any other country’s mobile data. Thus, it can reasonably represent all visitors to Jeju Island. Despite these unique advantages, SKT data has limitations, that is, it cannot provide information on each individual’s spatiotemporal movement because of personal privacy issues. Furthermore, while this study provides an empirical analysis of the effective use of mobile phone data, it lacks integration with crowdsourced geospatial data [35,47], which could provide a broader and more nuanced understanding of visitor dynamics. Future research should provide analytical methods that can be combined with heterogeneous sample data, such as geotagged social media data or crowdsourced datasets to provide richer insights. Research on the effects of climate change on tourism patterns is crucial for long-term tourism policy and planning. A multidimensional approach will expand the insights necessary for sustainable development practices in Jeju Island and other tourist destinations.
